# Associations between dietary fatty acids intake and abdominal aortic calcification: a national population-based study

**DOI:** 10.1186/s12944-024-02059-3

**Published:** 2024-03-09

**Authors:** Yan Xiao, Yingping Quan

**Affiliations:** 1 Department of Obstetrics, The Second Affiliated Hospital, Hengyang Medical school, University of South China, Hengyang, 421001 China; 2 Department of Gastrointestinal Surgery, The Affiliated Nanhua Hospital, Hengyang Medical school, University of South China, Hengyang, 421002 China

**Keywords:** Abdominal aortic calcification, Fatty acids, NHANES, Dietary intake

## Abstract

**Background:**

Abdominal aortic calcification (AAC) is a crucial indicator of cardiovascular health. This study aims investigates the associations between dietary fatty acid intake and AAC.

**Methods:**

In this study, a cross-sectional assessment was performed on a group of 2,897 individuals aged 40 and above, utilizing data from the NHANES. The focus was on examining dietary consumption of various fatty acids, including Saturated (SFA), Monounsaturated (MUFA), Polyunsaturated (PUFA), as well as Omega-3 and Omega-6. The evaluation of AAC was done by applying the Kauppila AAC score to results obtained from dual-energy X-ray absorptiometry scans. For statistical analysis, weighted multivariate linear and logistic regression were employed, with adjustments for variables like gender, age, ethnicity, and overall health condition.

**Results:**

Participants with higher intake of SFA and PUFA showed a positive association with AAC score, while higher levels of dietary Omega-3 and Omega-6 fatty acids was connected with a negative correlation. Subgroup analyses indicated consistent associations across different sexes and age groups. The study found that an increase in SFA and PUFA intake correlated with an increase in AAC score, whereas Omega-3 and Omega-6 intake correlated with a decrease.

**Conclusion:**

This study underscores the importance of dietary fatty acid composition in the prevalence of AAC and its potential implications for dietary guidelines and cardiovascular disease prevention strategies.

## Introduction

Abdominal aortic calcification (AAC), characterized by the abnormal deposition of minerals like calcium and phosphate within the abdominal aorta wall, serves as an early indicator of atherosclerotic calcification and independently predicts cardiovascular events and overall mortality, often preceding coronary artery calcification [1, 2]. Extensive studies have underscored intricate links of AAC to various medical conditions. For instance, a prospective cohort study highlighted that individual with severe AAC faced an elevated risk of developing dementia later in life [3]. In a retrospective case-control research, AAC was linked to the rupture of intracranial aneurysms [4]. Despite these significant correlations, effective treatments for AAC remain elusive. A small-scale randomized controlled trial showed that sodium thiosulphate could mitigate calcification in the iliac arteries and cardiac valves but did not effectively alleviate AAC in end-stage kidney disease patients [5]. Consequently, the prevention and management of AAC continue to be a formidable challenge, underscoring the need for further research in this area.

The dietary consumption of fatty acids, especially the intake of omega-3 and omega-6, has been a significant focus in cardiovascular health research [6, 7]. Insights from the NHANES indicate that elevated plasma levels of linoleic acid (an n-6 fatty acid) correlate with decreased adiposity and a lower incidence of metabolic syndrome, hinting at its beneficial role in preventing cardiometabolic diseases [8, 9]. Furthermore, dietary intake of Omega-3 and n-6 fatty acids has been inversely associated with the risk of hypertension, indicating their importance in cardiovascular health management [10]. Additionally, the intake of Omega-3 and n-6 fatty acids negatively correlates with hypertension risk, underscoring their relevance in cardiovascular wellness. Further research also links specific polyunsaturated fatty acid (PUFA) consumption patterns to mortality rates, with a noted association between dietary marine omega-3 PUFA and reduced mortality in some groups [11].

However, the relationship between these fatty acids and AAC specifically has not been thoroughly explored in the National Health and Nutrition Examination Survey (NHANES). Given the established links between fatty acid intake and cardiovascular risk factors, it is plausible to hypothesize a potential association with AAC development. The objective of this research is to explore how dietary fatty acid consumption correlates with the occurrence of AAC, leveraging the extensive dataset provided by the NHANES. This approach aims to bridge the knowledge gap in this area of study.

## Methods

### Study population

**Fig. 1:**
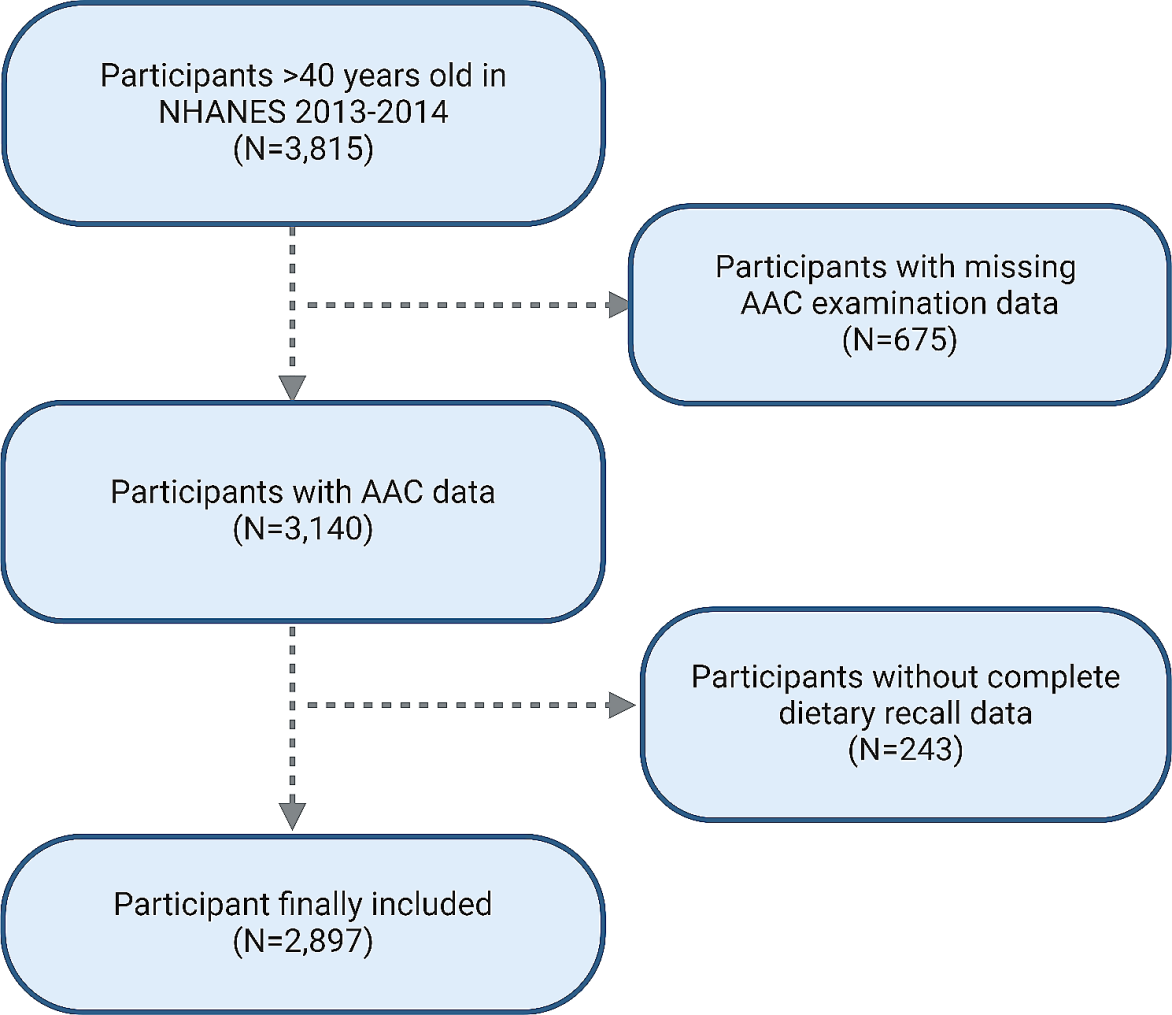
Flow chart of participants selection. NHANES, national health and nutrition examination survey

The NHANES, conducted by the National Center for Health Statistics, is a pivotal program that aims to assess the health and nutritional status of the U.S. population [12, 13]. It provides critical data on the prevalence of major diseases and associated risk factors through extensive data collection. NHANES employs sophisticated multi-stage, probability-based sampling methods to ensure a nationally representative sample [14]. The program’s protocols have received approval from the National Center for Health Statistics (NCHS) Research Ethics Review Board, and all participants provided written informed consent. This study utilized data from the 2013–2014 NHANES cycle. The study focused on individuals aged 40 years and older, as AAC data were not available for younger participants. From the initial pool of 10,175 individuals in the 2013–2014 NHANES cycle, the study excluded those under 40 years (*n* = 6,360), along with participants with missing AAC scores (*n* = 675) and incomplete dietary recall data (*n* = 243), resulting in a final sample of 2,897 participants for analysis (Fig. 1).

### Dietary fatty acids

In this study, the analysis of dietary fatty acids was carried out using the 24-hour dietary recall interviews from NHANES. This method is recognized for its accuracy in gathering comprehensive data on the consumption of various foods and drinks over the past 24 h, including specific fatty acids [15, 16]. The investigation primarily centered on the intake of Saturated Fatty Acids (SFA), Monounsaturated Fatty Acids (MUFA), Polyunsaturated Fatty Acids (PUFA), particularly Omega-3 and Omega-6. These fatty acids are key to understanding dietary habits and their implications for health, given their distinct roles in metabolism and disease risk factors.

### Abdominal aortic calcification

AAC in this research was evaluated using the Kauppila AAC score obtained from dual-energy X-ray absorptiometry (DXA) scans targeting the lateral lumbar spine. This scoring approach, widely acknowledged for its effectiveness, measures AAC severity, with higher scores representing more extensive calcification. The assessment involves dividing the abdominal aorta wall into four segments, each corresponding to L1-L4 vertebral regions. These segments receive scores from 0 to 6 based on calcium deposition levels. The overall AAC score, which can range from 0 to 24, is the cumulative score of these segments. For the purposes of this study, an AAC score above 6 was identified as a marker of severe AAC, aligning with the threshold commonly used in similar research [2, 17].

### Covariables

The selection of covariates for this study was guided by their potential influence on the connections between dietary fatty acid and AAC. The covariates included were sex, age, race, education level, total energy intake, diabetes status, hypertension status, use of hypertension medication, High-Density Lipoprotein Cholesterol (HDL-C), family Poverty Income Ratio (PIR), Body Mass Index (BMI), and total cholesterol levels. These covariates were chosen based on their established relevance in epidemiological research and their potential confounding effects on the study outcomes [18].

### Statistical analysis

To address missing data in the dataset, multiple imputation was utilized. This technique involves creating several different plausible imputed datasets and then averaging the results to account for the uncertainty caused by the missing data. The baseline characteristics of the study population were analyzed with continuous variables presented as mean and standard deviation. The ANOVA test was applied to compare these variables between groups. Categorical variables were expressed as percentages, with the Chi-square test determining their *P* values. The dietary fatty acids intake was categorized into quartiles based on their distribution within the study population. This categorization allowed for the assessment of trends and potential dose-response relationships between fatty acid intake levels and AAC. The study further examined the relationship between dietary fatty acid intake (including SFA, MUFA, PUFA, Omega-3, and Omega-6) and AAC scores, utilizing weighted multivariate linear regression. The investigation into the association of fatty acid intake with the prevalence of severe AAC was conducted through weighted multivariate logistic regression. These models were adjusted for various covariates to account for possible confounding factors. Subgroup analyses were conducted to investigate the relationship between dietary fatty acids intake and AAC across different demographic segments, particularly sex and age groups. This decision was informed by existing studies indicating significant variations in fatty acid levels among different sexes and age groups, which may alter the association with cardiovascular risk [19, 20]. Our analyses aimed to elucidate any differential associations between fatty acid consumption and AAC within these specific populations. The statistical analyses for this study were conducted using R software, version 4.2, chosen for its sophisticated statistical features and ability to manage complex survey datasets such as NHANES. Statistical significance was established at a two-sided *p*-value below 0.05 [21, 22]. This criterion was consistently applied in evaluating the significance of the links between dietary fatty acid intake and AAC, as well as in all other statistical tests in the research.

## Results

### Baseline characteristics

Among 2,897 participants were enrolled in this study. A total of 1,395 males and 1,502 females with a mean age of 58.6 ± 11.9 years were enrolled in these participants, of which 2,030 (70.7%) were without AAC, 553 (19.9%) were diagnosed with non-severe AAC and 314 (10.8%) were diagnosed with severe AAC (AAC score > 6).

**Fig. 2:**
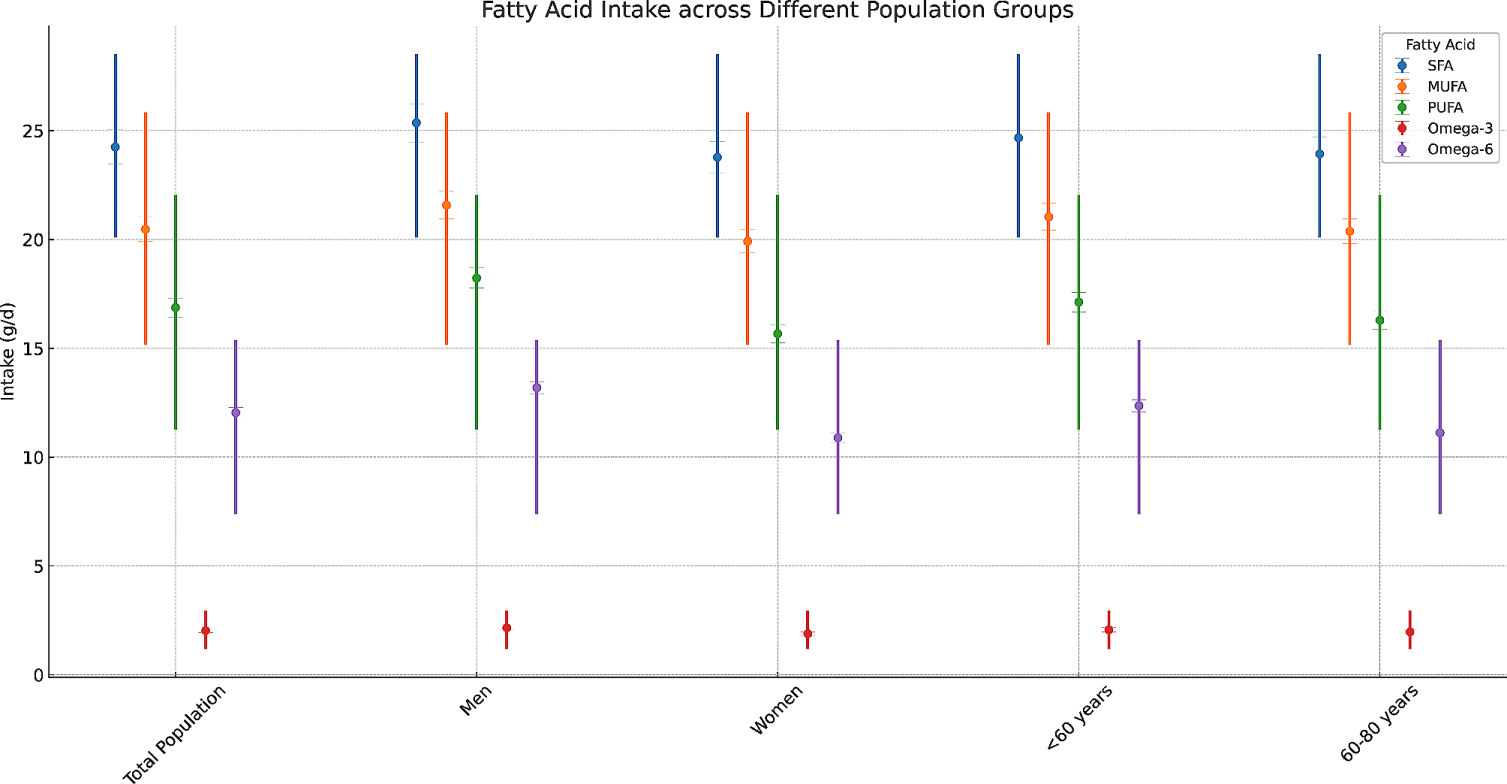
Distribution of fatty acids intake across different population groups. This figure illustrates the mean intake of various fatty acids (SFA, MUFA, PUFA, Omega-3, Omega-6) across different population groups including the total population, men, women, individuals under 60 years, and those between 60–80 years. Each fatty acid type is represented by a unique color and symbol, with error bars indicating the standard deviation around the mean intake. Additionally, solid lines denote the range of intake for each fatty acid, providing a comprehensive view of dietary patterns across these groups

Participants diagnosed with severe AAC had a higher proportion of non-Hispanic whites, a higher proportion of smokers, and individuals with hypertension and diabetes than participants with an AAC score less than or equal to 6; but had lower BMI and dietary intake levels (Table 1). Figure 2 depicts the average intake of various fatty acids (SFA, MUFA, PUFA, Omega-3, Omega-6) in different population groups.

**Table 1:** Basic characteristics of participants grouped by AAC

Characteristics	Non-AAC (*N* = 2,030)	Non-severe AAC (*N* = 553)	Severe AAC (*N* = 314)	*P*-value
Age (years)	56.00 ± 10.93	61.37 ± 11.67	70.99 ± 9.43	< 0.001
Gender, (%)				0.269
Male	966 (47.59%)	283 (51.18%)	146 (46.50%)	
Female	1064 (52.41%)	270 (48.82%)	168 (53.50%)	
Race/ethnicity, (%)				< 0.001
Non-Hispanic White	831 (40.94%)	280 (50.63%)	196 (62.42%)	
Non-Hispanic Black	430 (21.18%)	95 (17.18%)	46 (14.65%)	
Mexican American	293 (14.43%)	57 (10.31%)	27 (8.60%)	
Other races	476 (23.45%)	121 (21.88%)	45 (14.33%)	
Education level, (%)				0.071
< high school	445 (21.92%)	119 (21.52%)	78 (24.84%)	
High school	434 (21.38%)	136 (24.59%)	80 (25.48%)	
> high school	1151 (56.70%)	298 (53.89%)	156 (49.68%)	
Drinking alcohol, (%)				0.969
Yes	549 (28.23%)	149 (27.90%)	221 (71.29%)	
No	1396 (71.77%)	385 (72.10%)	89 (28.71%)	
Diabetes, (%)				< 0.001
Yes	375 (18.47%)	114 (20.61%)	113 (35.99%)	
No	1655 (81.53%)	439 (79.39%)	201 (64.01%)	
Smoking, (%)				< 0.001
Yes	863 (42.51%)	285 (51.54%)	186 (59.24%)	
No	1167 (57.49%)	268 (48.46%)	128 (40.76%)	
Hypertension, (%)				< 0.001
Yes	854 (42.07%)	294 (53.16%)	233 (74.20%)	
No	1176 (57.93%)	259 (46.84%)	81 (25.80%)	
Take medicine for hypertension, (%)				< 0.001
Yes	554 (27.29%)	215 (38.88%)	223 (71.02%)	
No	1476 (72.71%)	338 (61.12%)	91 (28.98%)	
Energy (kCal/d)	2048.00 ± 966.93	2062.46 ± 984.27	1742.54 ± 784.24	< 0.001
Total saturated fatty acids (g/d)	24.86 ± 16.25	25.76 ± 16.76	22.04 ± 12.51	0.043
Totalmonounsaturated fatty acids (g/d)	27.47 ± 16.99	28.78 ± 19.40	24.23 ± 13.87	0.003
Total polyunsaturated fatty acids (g/d)	18.51 ± 12.59	19.09 ± 13.41	16.77 ± 11.24	0.017
Omega-3 fatty acids intake (g/d)	1.67 ± 1.01	1.58 ± 1.13	1.46 ± 1.09	0.003
Omega-6 fatty acids intake (g/d)	12.93 ± 9.05	12.11 ± 9.23	9.96 ± 9.46	< 0.001
Family PIR	2.73 ± 1.67	2.65 ± 1.63	2.56 ± 1.53	0.332
BMI	28.88 ± 5.87	28.05 ± 5.07	27.20 ± 4.37	< 0.001
Total cholesterol (mg/dL)	196.51 ± 44.34	195.63 ± 44.32	183.91 ± 41.16	< 0.001
HDL-C (mg/dL)	54.55 ± 17.06	52.57 ± 14.94	53.14 ± 16.34	0.114
AAC score	0.58 ± 1.26	0.58 ± 1.26	10.41 ± 4.04	< 0.001

Mean ± SD for continuous variables: The *P* value was calculated by the ANOVA test; (%) for categorical variables: The *P* value was calculated by the Chi-square test. Abbreviation: PIR, the ratio of income to poverty, BMI, body mass index; Q, quartile; HDL-C, high-density lipoprotein; AAC, abdominal aortic calcification

### Association between dietary fatty acids and AAC

Table 2 presents the associations between dietary fatty acid intake (SFA, MUFA, PUFA, Omega-3, and Omega-6) and AAC scores, adjusted for all covariates. There were observed associations indicating that increased intake of SFA and PUFA was associated with an incremental effect on AAC scores, although the association for MUFA did not reach statistical significance. Specifically, an increase of 1 g per day in SFA and PUFA intake was associated with changes in AAC scores, as evidenced by effect sizes of 0.01 [95% CI: 0.01, 0.03] for SFA and 0.02 [95% CI: 0.00, 0.03] for PUFA, respectively. Conversely, higher intakes of Omega-3 and Omega-6 fatty acids were associated with a decrease in AAC scores, with effect sizes of -0.05 [95% CI: -0.08, -0.02] for Omega-3 and − 0.09 [95% CI: -0.12, -0.06] for Omega-6, indicating beneficial associations. Furthermore, participants with higher intakes of Omega-3 and Omega-6 exhibited significant reductions in AAC scores by 0.61 and 0.88 points, respectively, compared to those with lower intakes (*P* for trend < 0.05).

**Table 2:** Multivariate regression analyses of the associations between dietary fatty acids and AAC

Fatty acids	Continuous	Q1	Q2	Q3	Q4	*P* for trend
(g/d)	β/OR (95% CI)		β/OR (95% CI)	β/OR (95% CI)	β/OR (95% CI)	
**AAC score**						
Total SFAs	0.01 (0.01, 0.03)*	0 (ref)	0.11 (-0.26, 0.48)	0.28 (-0.11, 0.68)	0.32 (-0.19, 0.83)	0.189
Total MUFAs	0.01 (-0.00, 0.03)	0 (ref)	-0.01 (-0.37, 0.36)	0.16 (-0.24, 0.56)	0.25 (-0.26, 0.76)	0.271
Total PUFAs	0.02 (0.00, 0.03)*	0 (ref)	0.18 (-0.18, 0.55)	0.33 (-0.06, 0.72)	0.44 (-0.03, 0.90)	0.066
Omega-3 FA	-0.05 (-0.08, -0.02)*	0 (ref)	-0.11 (-0.17, -0.05)*	-0.26 (-0.47, -0.07)*	-0.61 (-1.07, -0.15)*	0.020*
Omega-6 FA	-0.09 (-0.06, -0.03)*	0 (ref)	-0.18 (-0.26, -0.09)*	-0.35 (-0.55, -0.16)*	-0.88 (-1.52, -0.25)*	0.004*
**Severe AAC**						
Total SFAs	1.01 (1.00, 1.02)*	1 (ref)	1.16 (0.77, 1.76)	1.58 (1.01, 2.48)	1.82 (0.99, 3.34)	0.036*
Total MUFAs	1.01 (1.00, 1.03)*	1 (ref)	1.16 (0.78, 1.73)	1.43 (0.90, 2.27)	1.44 (0.78, 2.68)	0.215
Total PUFAs	1.02 (1.00, 1.04)*	1 (ref)	1.27 (0.86, 1.87)	1.46 (0.95, 2.27)	1.63 (0.96, 2.77)	0.071
Omega-3 FA	0.98 (0.97, 0.99)*	1 (ref)	0.90 (0.82, 0.98)*	0.81 (0.62, 1.00)	0.76 (0.55, 0.97)*	0.033*
Omega-6 FA	0.96 (0.93, 0.99)*	1 (ref)	0.87 (0.79, 0.95)*	0.65 (0.40, 0.91)*	0.50 (0.28, 0.78)*	< 0.001*

Models were adjusted for sex, age, race, education, energy, diabetes, hypertension, hypertension medication, BMI, HDL-C, family PIR, and total cholesterol. Continuous, Ln-transformed concentration of fatty acids; Q, quartile; 95% CI, 95% confidence interval; PIR, the ratio of income to poverty, BMI, body mass index; HDL-C, high-density lipoprotein; AAC, abdominal aortic calcification; SFA, saturated fatty acid; MUFA, monounsaturated fatty acid; PUFA, polyunsaturated fatty acid. * indicates *p* < 0.05, indicating statistical significance

The associations between dietary fatty acid (SFA, MUFA, PUFA, Omega-3, and Omega-6) intake and severe AAC were similar. there was a positive association between the three major dietary fatty acid intakes and severe AAC, and a negative association between Omega-3 and Omega-6 intake and severe AAC. Specifically, for every 1 g per day increase in intake, the odds of severe AAC increased by ORs of 1.01 for SFA, 1.01 for MUFA, and 1.02 for PUFA, indicating a 1%, 1%, and 2% increase in the odds of severe AAC, respectively. Conversely, Omega-3 and Omega-6 intake were associated with decreased odds of severe AAC, with ORs of 0.98 and 0.96, respectively, corresponding to a 2% and 4% reduction in the odds of severe AAC. Participants with higher intake of Omega-3 and Omega-6 exhibited significantly lower odds of severe AAC, with ORs of 0.76 [95% CI: 0.55, 0.97] and 0.50 [95% CI: 0.28, 0.78], respectively (*P* for trend < 0.05).

### Subgroup analyses

Subgroup analyses based on sex and age further demonstrated the robustness of the association between dietary fatty acid intake and AAC across populations (Table 3). The associations between dietary fatty acids and AAC score and severe AAC were consistent across all subgroups: positive associations between SFA, MUFA and PUFA intake and AAC, and negative associations between Omega-3 and Omega-6 intake and AAC. However, it is worth mentioning that in the different age subgroups (< 60 years and 60–80 years), although the negative association between Omega-3 and Omega-6 intake and AAC remained, the negative effect of Omega-3 and Omega-6 intake on the odds of severe AAC was significantly greater in the 60–80 year olds than in the < 60 year olds (*P* for interaction < 0.05).

**Table 3:** Subgroup analyses of the associations between dietary fatty acids and AAC

Fatty acids	Sex [β/OR (95% CI)]			Age [β/OR (95% CI)]		
(g/d)	Men	Women	*P* for interaction	< 60 years	60–80 years	*P* for interaction
**AAC score**						
Total SFAs	0.01 (0.01, 0.03)*	0.01 (0.00, 0.02)*	0.561	0.01 (0.01, 0.03)*	0.02 (-0.01, 0.04)	0.385
Total MUFAs	0.01 (0.00, 0.03)	0.01 (0.01, 0.02)*	0.682	0.01 (0.01, 0.03)*	0.01 (0.01, 0.02)*	0.699
Total PUFAs	0.01 (-0.00, 0.03)	0.02 (0.01, 0.03)*	0.209	0.01 (-0.00, 0.02)	0.02 (0.01, 0.04)*	0.310
Omega-3 FA	-0.06 (-0.09, -0.03)*	-0.04 (-0.07, -0.01)*	0.098	-0.05 (-0.09, -0.01)*	-0.07 (-0.11, -0.03)*	0.055
Omega-6 FA	-0.08 (-0.12, -0.04)*	-0.09 (-0.16, -0.02)*	0.236	-0.07 (-0.11, -0.04)*	-0.10 (-0.16, -0.03)*	0.046*
**Severe AAC**						
Total SFAs	1.01 (1.00, 1.02)*	1.01 (1.00, 1.03)*	0.188	1.02 (1.01, 1.03)*	1.01 (1.00, 1.03)*	0.342
Total MUFAs	1.01 (1.00, 1.03)*	1.01 (1.00, 1.03)*	0.872	1.01 (1.00, 1.03)*	1.02 (1.00, 1.04)*	0.481
Total PUFAs	1.02 (1.00, 1.04)*	1.02 (1.01, 1.03)*	0.653	1.03 (1.01, 1.04)*	1.02 (1.01, 1.03)*	0.098
Omega-3 FA	0.99 (0.97, 1.00)	0.98 (0.97, 0.99)*	0.212	0.99 (0.98, 1.00)*	0.95 (0.92, 0.98)*	0.003*
Omega-6 FA	0.95 (0.92, 0.99)*	0.97 (0.94, 0.99)*	0.070	0.96 (0.93, 0.99)*	0.92 (0.87, 0.98)*	< 0.001*

Models were adjusted for sex, age, race, education, energy, diabetes, hypertension, hypertension medication, BMI, HDL-C, family PIR, and total cholesterol. Continuous, Ln-transformed concentration of fatty acids; Q, quartile; 95% CI, 95% confidence interval; PIR, the ratio of income to poverty, BMI, body mass index; HDL-C, high-density lipoprotein; AAC, abdominal aortic calcification; SFA, saturated fatty acid; MUFA, monounsaturated fatty acid; PUFA, polyunsaturated fatty acid. * indicates *p* < 0.05, indicating statistical significance

## Discussion

Our study involving 2,897 representative adult NHANES participants showed associations between dietary fatty acids and AAC. We observed positive associations between increased intake of the three major classes of dietary fatty acids (SFA, MUFA, PUFA) and AAC, whereas Omega-3 and Omega-6 intake were negatively associated. These findings suggest a potential role for dietary fatty acids in the regulation of vascular health, particularly with respect to AAC, which is known to predict cardiovascular disease morbidity and mortality.

### Comparison with previous studies

As far as our knowledge extends, this study represents the inaugural exploration of the relationship between various dietary fatty acids and AAC within a US population. Previous research has predominantly focused on examining the connection between specific types of fatty acids and cardiovascular disease (CVD). A study based on the Melbourne Collaborative Cohort Study (*n* = 312) investigated the association of several subcategories of dietary fatty acid intake with AAC and showed that alpha-linolenic acid (ALA) and Omega-3 intake reduced the risk of AAC, but the association was limited to older women but not men [23]. However, such differences may not be real, and our results support the idea that higher levels of dietary Omega-3 and Omega-6 are connected with lower AAC scores in both older men and women. Smaller sample sizes and regional, population-based differences may account for much of the variation in results. A study in several European countries investigated the association between SFA, MUFA, and PUFA intake and coronary heart disease (CHD), and although the findings demonstrated that dietary fatty acid intake was not connected with CHD, an association between SFA intake and CHD could be found if the associations were broken down to the level of intake of the different substances [24]. The potential relationship between dietary intake of Omega-3 and Omega-6 with the risk of CVD has also been extensively investigated, and most studies support our results that there is a negative association between Omega-3 and Omega-6 intake and the risk of CVD [25]. A multiethnic cohort study demonstrated an inverse association between dietaryOmega-3 and Omega-6 and CVD incidence, and this study also showed that circulating fatty acid levels were also significantly negatively associated with CVD risk [26].

### Potential biological mechanisms

The positive correlation between SFA, MUFA, PUFA intake, and AAC can be attributed to several mechanisms. SFAs are known to induce endothelial dysfunction and promote a pro-inflammatory state, which accelerates vascular calcification, a key component of AAC [27]. MUFAs, while generally considered healthier, can also contribute to AAC in excessive amounts by altering lipid profiles and potentially enhancing lipid oxidation, leading to vascular damage [28]. PUFAs, particularly certain omega-6 fatty acids, may promote inflammation through the production of pro-inflammatory eicosanoids, which have been linked to the development of vascular calcification [29]. In contrast, omega-3 fatty acids, includes EPA and DHA, exhibit protective effects against AAC by reducing inflammation and inhibiting the expression of osteogenic markers in vascular cells, thereby mitigating the calcification process [30, 31]. Additionally, omega-6 fatty acids, in a balanced ratio with omega-3, can contribute to this protective effect by modulating inflammatory responses and improving lipid metabolism [32, 33]. In addressing the differential effects of omega-3 and omega-6 fatty acids on AAC, recent studies shed light on their distinct roles in lipid metabolism and vascular health. Omega-3 fatty acids, particularly EPA and DHA, have been shown to exhibit anti-inflammatory properties that may protect against vascular calcification and the progression of conditions such as abdominal aortic aneurysm (AAA) [34]. Conversely, omega-6 fatty acids, such as arachidonic acid, have been associated with an increased presence and progression of AAA, potentially through pro-inflammatory pathways [35]. These findings suggest that while omega-3 fatty acids may confer protective effects against vascular calcification, omega-6 fatty acids could have adverse effects under certain conditions. This apparent contradiction highlights the complex role of fatty acids in lipidemic metabolism and their impact on cardiovascular health.

### Strengths and limitations

The primary advantage of our research lies in its utilization of the NHANES, which employs a multi-stage probability sampling design. This approach enhances the reliability and robustness of these findings, ensuring that they reflect the broader U.S. population. Additionally, our study’s comprehensive assessment of dietary fatty acid intake and its association with AAC provides valuable insights into potential dietary interventions for AAC management and prevention. We also addressed the dose-response relationship between different types of fatty acids and AAC, identifying minimal thresholds for their beneficial or detrimental associations. However, several limitations must be acknowledged. Firstly, the cross-sectional nature of our study precludes establishing a causal relationship between dietary fatty acid intake and AAC [36]. While we controlled for a range of potential confounding variables, the possibility of residual confounding cannot be entirely ruled out [37]. Longitudinal studies are required to confirm the causal nature of the observed associations. Secondly, the reliance on self-reported dietary intake data, despite being detailed and comprehensive, may be subject to recall bias and estimation errors. This limitation underscores the need for more objective dietary assessment methods in future research. Finally, while our study is representative of the U.S. adult population aged 40 and above, the generalizability of our findings to younger individuals or populations from different regions remains unknown. Further research in diverse populations is necessary to validate and extend our findings.

## Conclusion

In conclusion, our study contributes valuable insights into the role of dietary fatty acids in AAC, with implications for dietary guidelines and public health strategies.

## Data Availability

No datasets were generated or analysed during the current study.

## Abbreviations

AAA: Abdominal aortic aneurysm
AAC: Abdominal aortic calcification
NHANES: National Health and Nutrition Examination Survey
CVD: Cardiovascular disease
SFA: Saturated Fatty Acids
MUFA: Monounsaturated Fatty Acids
PUFA: Polyunsaturated Fatty Acids
NCHS: National Center for Health Statistics
DXA: Dual-energy X-ray absorptiometry
BMI: Body mass index
HDL-C: High-density lipoprotein cholesterol
PIR: Income-to-Poverty Ratio
CHD: Coronary heart disease
